# Medical professionalism: what the study of literature can contribute to the conversation

**DOI:** 10.1186/s13010-015-0030-0

**Published:** 2015-06-27

**Authors:** Johanna Shapiro, Lois L. Nixon, Stephen E. Wear, David J. Doukas

**Affiliations:** Family Medicine and Director of the Program in Medical Humanities & Arts, University of California-Irvine, School of Medicine, 101 City Dr. South, Rte 81, Bldg 200, Ste 835, Orange, CA 92868 USA; Internal Medicine, Division of Ethics and Humanities, University of South Florida School of Medicine, 12901 Bruce B Downs Blvd, Tampa, FL 33612 USA; Center for Clinical Ethics and Humanities in Healthcare, Departments of Medicine, Gynecology-Obstetrics, and Philosophy, University at Buffalo SUNY School of Medicine, Buffalo, NY USA; William Ray Moore Endowed Chair of Family Medicine and Medical Humanism, and Division of Medical Humanism and Ethics, Department of Family and Geriatric Medicine, University of Louisville, 2301S 3rd St, Louisville, KY 40292 USA

**Keywords:** Medical professionalism, Professional identity formation, Literature, Health humanities, Medical humanities

## Abstract

Medical school curricula, although traditionally and historically dominated by science, have generally accepted, appreciated, and welcomed the inclusion of literature over the past several decades. Recent concerns about medical professional formation have led to discussions about the specific role and contribution of literature and stories. In this article, we demonstrate how professionalism and the study of literature can be brought into relationship through critical and interrogative interactions based in the literary skill of close reading. Literature in medicine can question the meaning of “professionalism” itself (as well as its virtues), thereby resisting standardization in favor of diversity method and of outcome. Literature can also actively engage learners with questions about the human condition, providing a larger context within which to consider professional identity formation. Our fundamental contention is that, within a medical education framework, literature is highly suited to assist learners in questioning conventional thinking and assumptions about various dimensions of professionalism.

## Introduction

Over the past fifty years the study of literature has become a generally accepted aspect of medical education. As thoughtful scholars have recently considered how to teach professionalism effectively and meaningfully, questions have arisen about the role of stories, essays, first-person narratives, and poetry in facilitating the professional identity formation of medical students. Those who argue affirmatively imply that exposing students to literature will inculcate professionalism virtues and attributes [1]. Those who disagree assert that the study of literature has goals and purposes unrelated to professionalism [2]. In this article, we investigate definitions of medical professionalism, and frame its inclusion in the competency framework as an effort to anchor its abstract virtues in behavioral specificity. Next we consider how literature can advance our understanding of medical professionalism through a different kind of singularity grounded in the literary method of close reading. Ultimately, we contend that the development of medical professionalism will benefit from the critical and interrogative methods of literature.

This article is a result of the Project to Rebalance and Integrate Medical Education (PRIME), sponsored by the Patrick and Edna Romanell Foundation. PRIME focused on how medical ethics and humanities education are prerequisite to professionalism formation in medical school and residency training [3, 4]. PRIME, in turn, resulted in the creation of the Academy for Professionalism in Health Care as an organization devoted to professionalism education [5].

### The conundrum of professionalism in medical education

There are at least two significant issues to consider in discussing medical professionalism. One has to do with the *content* of professionalism itself, i.e., how it is defined. The second is essentially an implementation issue, i.e., the *methods* which establish how professionalism is achieved. These issues, and their implications for professionalism education, are discussed below.

### Defining medical professionalism

The Medical Professionalism Project initiated by the American Board of Internal Medicine Foundation, the American College of Physicians Foundation, and the European Federation of Internal Medicine resulted in a professionalism charter consisting of virtue-based attributes such as altruism, trust, honesty, patient empowerment, and commitment to social justice [6]. Medical educatorshave also argued for a virtue-based definition, including qualities of compassion, integrity; truth-telling; respect for others; self-effacement; and fidelity to patients [7–10]. Prior PRIME publications acknowledged the importance of scientific and clinical *competence* using established rigorous evidence-based medicine; while emphasizing *promotion* of patients’ best interests as the clinician’s primary moral consideration (with self-interest as a subservient claim) and honoring the exercise of the *public trust*, as a necessary obligation to carry forth the fiduciary traditions of medicine (as opposed to guild-like self-interest) [4]. Other definitions also support the commitment to and reinforcement of moral values and ethical principles [11, 12].

These definitions, while valuable, highlighted primarily general, abstract virtues and attributes that have proved difficult to translate into daily actions. Recent considerations of professionalism and professional identity formation have stressed the necessity of moving from abstraction to practice [13, 14], highlighting what is often referred to as phronesis or practical wisdom [15]. Medical educators have wrestled with this challenge for the past decade, most notably through the effort to incorporate medical professionalism into the competency framework.

### Professionalism as a competency

Indeed, it could be argued that the rise of the competency movement in medical education [16] has been an effort to anchor generalities of training in specific, concrete, measurable behaviors. In terms of professionalism specifically, attempting to inculcate values and virtues often struck both learners and educators as threatening and potentially implying character defects in students [17]. Thus, professionalism moved from the conceptual realm to become one of six essential medical education competencies, sometimes viewed as a “meta”- or “ordering” contextual competency for more technical competencies [18, 19]. In this respect, competency-based education appeared to offer a “solution” to the abstract nature of earlier approaches to conceptualizing professionalism, precisely because of its behavioral specificity. Many medical educators found the notion of professional competencies appealing because they seemed to offer the promise of transforming amorphous, ill-defined, and difficult-to-measure qualities into instrumental behaviors that were observable and assessable. Recently more detailed “milestones” have been added to supplement and refine the six competencies, but these remainrooted in the establishment of measurable behavior [20]. Whether discussing milestones or competencies, the language employed reflects a tendency in these guidelines to prescribe, control, and shape learners in specific, reductive directions.

### Challenging a behavioral approach to medical professionalism

Even as professionalism became identified as an area of medical competence, some medical educators’ reflections on the topic continued to reveal a discomfort with behavioral pedagogical approaches, instead advocating for developing, reinforcing, and sustaining deeply held attitudes and values [17, 21]. As Hanna and Fins write, medical students must learn how to “*be* good doctors, rather than merely to act like good doctors [18, 22, 23]”.

Others also assert that behavioral professionalism tempts students to behave in ways that fulfill others’ expectations of professionalism without actually believing in the virtues or principles that underpin these behaviors [24], resulting in an emphasis on surface impression management [25]. Others complain that in clinical settings, professionalism is simplistically and narrowly defined as a technical problem, with most solutions offered being prescriptive, mechanical, and rule-bound [26].

### Setbacks in teaching professionalism

With some notable exceptions, such as small group reflection-based sessions [27, 28] that have shown promise most approaches to teaching professionalism, implicitly or explicitly rooted in the competency model, have not documented significant success. An article by two medical students claims that medical educators are more likely to evaluate appearance, formality, and conformity as “professional” than they are to pay attention to traits of honor, altruism, and responsibility. This “view from the trenches” suggests that adherence to hospital etiquette, respecting academic hierarchy, and subservience to authority are valued more than patient-centered virtues [29]. A survey study examining student attitudes toward professionalism found that almost a third of respondents felt professionalism education was patronizing and demeaning [30], while a more in-depth qualitative study concluded that medical students made a distinction between “good” doctors and “professional” doctors, and perceived professionalism as an external and imposed construct [31].

One troubling study found that, despite required professionalism training, unprofessional behavior in students actually *increased* during their clinical years [32]. These and similar concerns suggest that students see professionalism training as little more than a tool of governance [33] wielded by supervisors promoting exterior and often trivial performance, rather than emphasizing virtue.

The dilemma is clear. Medical educators have agreed to define professionalism as a competency to be achieved by measurable behaviors. They simultaneously recognize it to be a deeper, more meaningful sense of identity that incorporates a set of humanistic attitudes, behaviors, and critical thinking skills. Some medical educators hope that the study of literature can help resolve this educational impasse by contributing a new perspective to our understanding of professionalism that, like the competency model, attempts to bridge the gap between theory and practice, but does so in a radically different way. Our argument is that the study of literature is where we learn, in an emotionally and critically engaged way, to see how characters face moral dilemmas, how they resolve them, and the consequences of those resolutions.

## Implications of the Study of Literature for Medical Professionalism

If competencies have not provided a meaningful format for teaching medical professionalism, nevertheless it is a fundamental contention of the PRIME scholars that professionalism must involve the application of virtues to the *practice* of medicine [34]. We believe that the study of literature, with its emphasis on the discreteness of specific texts, has an important role to play in assisting learners in professionalism formation. One crucial way in which this occurs is by developing in learners the habit of close reading, a fundamental literary skill.

### How close reading relates to medical professionalism

Close reading has been defined as a disciplined reading and rereading of complex texts to identify layers of meaning that lead to more nuanced interpretation and deeper, more subtle understanding [35]. It is not difficult to imagine the translational relevance of close reading for developing a meaningful medical professionalism tied to the particulars of each patient’s care. Like a patient encounter, close reading first requires attentive observation – what does the reader notice about the text? What does the doctor notice about the patient? Interpretation follows observation – what is the *meaning* of the reader’s – or the doctor’s - observations [36]? Close reading requires a wariness of superficial and facile interpretations, a clinical position that helps the clinician avoid bias, assumptions, and judgmentalness.

A fundamental premise of close reading is the revisiting of texts to investigate alternative or complementary meanings while recognizing that there are not necessarily any right answers. Similarly, physicians trained in close reading may be more likely to continue to think about their patients and to remain open to new interpretations of their actions and attitudes. In close reading, students must not only “feel” a certain way in response to the text, but they must know how to defend their conclusions through reference to particular words and passages [37]. In the clinical context, physicians must be ready to question their initial emotional responses to patients in favor of more nuanced and complex responses that are based in evidence emerging from the clinical encounter.

Close reading interrogates the structure of a particular narrative. Why is a story told in a certain way? Who is telling the story? Who else might tell this story? How might different tellings change the nature of the story? Who is the intended audience for this story? Why are certain words selected and not others? Why are certain metaphors employed? What seems to be important or striking in the story? Are there contradictions or discrepancies in the story? Is the author trying to persuade the listener of something? What has been omitted from the story? Are there repetitions? What is the predominant tone of the story? Does it shift, and if so why? What patterns emerge in the text [38]? Such an approach, translated into the clinical encounter, is likely to result in a critical professionalism through respect, engaged attention, and critical thinking within a very specific context.

The implications of close reading for medical professionalism are far-reaching. In the remainder of this article, we discuss how close reading leads to a different and more critical way of understanding medical professionalism that is grounded in the specifics of each clinical encounter as well as the contextual specifics of race, gender, culture, and history. It is a method that questions conventional thinking about professionalism, complicates accepted virtues, and emphasizes individual variation.

### Asking meaningful questions rather than inculcating behavior

Although some scholars have suggested that studying literature can help medical students learn to better attend to and understand their patients’ stories [39], cultivate emotional resonance in patient care [40, 41], and address burn-out through supporting more examined, fulfilled professional lives [42], no educational process can guarantee or compel virtues, self-awareness, or wellbeing in learners. In the real world, medical educators are not always certain how such ineffable qualities or attributes can be meaningfully “demonstrated”. In these circumstances, what literature can do is help learners engage in critical thinking about *what* the virtues and values of medical professionalism might be; and *how* these actually might occur in particular situations influenced by culture, race, disability, gender, sexual orientation, and historical consideration.

Many professionalism issues are complicated, convoluted, and resist a simple behavioral solution (e.g., maintaining eye contact, touching a shoulder, employing rote expressions of empathy ). Rather, questions about how to think, feel, and behave professionally in a given circumstance are best approached as complex conundrums in which there will likely be disagreement among those involved about the nature of the problem, the desired resolution (if any), and the steps required to achieve it [26]. Studying literature can help prepare learners to grapple with these situations because stories suggest various responses without dictating them, urge consideration of different behaviors without ordering them, and illuminate values without oversimplifying them. Such approaches offer learners methods for exploring professionalism values that honor the distinctive, irreducible human qualities of each patient and each circumstance embedded in larger social and cultural contexts [43].

### The countercultural perspective

Although competencies by definition require instrumental goals, literary scholars are generally more comfortable advocating non-instrumental aims for the role of literature in medical education. One such overarching aim is the cultivation of a critical and questioning attitude toward conventional wisdom, a so-called “countercultural” [44] perspective on medicine that implicates both personal and professional moral development while situating medicine within a larger sociocultural framework [45, 46]. In this view, integrating literature into the curriculum should not blindly support the status quo in medicine, but instead should help learners question their own and the system’s preconceptions and prejudgments [47] to make transparent the values, culture, and ideology of medicine [48].

Drawing on critical theory, many health humanities scholars call for literature to open a “discursive space” that critiques conventional assumptions about medicine and the healthcare system [49, 50]. Dror argues that teaching literature offers a way of rethinking medicine, not instilling standards [48]. This approach emphasizes “catalyz[ing] emancipatory insights” [51] and creating an environment of “sustained critical reflection [52]”. Engaging with literature will not produce a set of measurable professionalism-specific behaviors in learners, but it is well-suited to facilitating a critical consciousness of self, others, and the world [53]. By stimulating critical thinking, literature enables learners to question established ways of understanding relationships between doctors and patients, doctors and other healthcare professionals and staff, and doctors and society. This standpoint asserts that, properly executed, literature should provoke discomfort and resistance in learners and disrupt their reflexive participation in healthcare [54, 55]. Kumagai and Wear call this process “making strange” taken-for granted assumptions and beliefs that may compromise humanistic care [56].

### Developing moral imagination

One way in which the study of literature can result in productive discomfort for students (and teachers!) is by critically interrogating the *meaning* of professionalism itself. Is professionalism primarily about protecting the “guild” of medicine? Is it about endorsing adherence to abstract virtues? Does it have to do with translating virtuous concepts into observable and measurable behaviors? Is it about a moral relationship between two (or more – often many more) people under trying circumstances? Working with a wide range of literary texts in a medical education context can help learners discover how to frame such questions and debate different answers.

Precisely how this happens is not fully circumscribed, but some scholars have argued that in part students become adept at both asking questions and exploring answers through the development of moral imagination, defined by Carson [57]as the heightened capacity to envision experience, whether one’s own or someone else’s, from a different perspective. Importantly, examination of literary texts reveals that in any given situation there are multiple ways of understanding and prioritizing events, thus making the privileging of any one perspective suspect. Charon refers to this as the capacity to visualize others’ narrative worlds [58]. Appreciation of differing points of view engages critical thinking through honing learner awareness of different, often contradictory but co-existing understandings [46]. It also facilitates empathetic orientation by encouraging emotional connection with or recognition of characters different from oneself and health-related roles different from one’s own [59, 60].

In discussing moral imagination, the psychiatrist Robert Coles [61] observes that stories admonish us, point us in new directions, and sometimes inspire us to lead lives of greater moral integrity. We should note that such aspirations are quite different from acquisition of standardized behaviors to be performed regardless of the particular situation and circumstance. Rather, selected stories stimulate moral imagination in medical learners by enabling them to step back from and become critically aware of their own values, beliefs, and assumptions about professionalism and how these are influenced by the dominant culture and other systems of influence in which they participate. From this beginning, learners can then imagine new possibilities for attitudes and action based on consideration of others’ values, perspectives, and priorities, especially those of disempowered and marginalized individuals, as well as their own. The critical thinking that emerges from the study of literature can help medical learners evaluate from a moral point of view both their original assumptions and dominating models of what professionalism is, as well as new possibilities they now envision in collaboration with their patients from a wider social perspective [62].

### The complication of professionalism values

Studying literature and reading stories reveal that even such enshrined professionalism values as compassion do not necessarily always serve moral ends; and point out ways in which such values need to be interrogated more critically to understand how they might go astray. Apparently beneficial qualities such as empathy, the ability to engender trust, and good communication skills all can be employed to encourage docility and compliance in less powerful individuals (i.e., patients) [2]. Some scholars have criticized the empathic skills trained in medical school for their potential as a tool to manipulate care, rather than as a virtue of care [63–65]. Similarly, compassion may devolve into a patronizing and demeaning position that approaches pity when not carefully and respectfully placed within the context of understanding the patient’s subjective experience of suffering within her culture, personal history, and values. Respect can be undermined through a mindless allegiance to autonomy in which physicians essentially abandon patients and families by expecting them to make medical decisions for which they have not been sufficiently prepared. Altruism can deteriorate into rigid self-sacrifice in physicians who think patients’ wellbeing requires a persistent neglect of personal wellbeing and life balance. By encouraging awareness of such nuances, reading literature critically and thoughtfully has the intriguing capacity to both challenge and deepen the virtues and attributes that comprise medical professionalism

### Standardization of professionalism?

The National Board of Medical Examiners calls for the “standardization” of professionalism in medicine [66]. From a literary perspective, with its emphasis on multiple, often contradictory perspectives and the importance of acknowledging the specifics of every situation, a “standardized” approach to professional attitudes, behaviors, and identity may not be possible. While elements of both standardization and diversity are likely important in formulating sufficiently complex views of professionalism [67], literature’s forte is to challenge “standardized” views of professionalism by invoking nuance and context. The role of literature is to cultivate a thoughtful examination of the implications and consequences of a spectrum of *different* attitudes, behaviors, and identities; and to situate these within a larger socioeconomic, cultural, and political context of power and privilege. Recalling Hanna and Fins’ concerns, we believe that literature offers a way to help students understand what it means to be, rather than merely act like, a humane professional. In this way, literature urges the opposite of “one size fits all” standardization by emphasizing the intrinsic value of diversity in how professionalism manifests filtered through each unique interaction of individuals (doctors, medical team, patients, and families), circumstances, and dominant discourses.

### Widening the lens

Competency involves standardized achievement of “correct” behaviors, a necessary narrowing to obtain reliability and consistency of assessment. Literature, on the other hand, offers a plethora of models and possibilities for being in the world and eschews the one right answer. Instead, the study of literature leads learners in directions that are open-ended, unpredictable, and self-determining. It can both widen the lens and provide insight into the complexities of the human condition, suffering, personhood, and our responsibility to each other [68]. Instead of compelling learners to narrow their focus to concrete behaviors, literature can help them realize that professionalism cannot be separated from an understanding of their own humanity and that of their patients. This is why students may learn more about professionalism from reading *War and Peace* than from an ACGME manual on professionalism milestones.^1^

### Assessment of professionalism

In contemplating the influence of the study of literature on students’ understanding of medical professionalism, how do we ascertain whether learners have actively engaged with what this concept might mean for them personally in different clinical situations? How do we achieve insight into what capacities and habits of mind they have developed as a result of their studies? In medicine, assessment approaches are often quantitative and numerical. Such an approach has little to propose in determining what happens to students as a result of critically reading a story or writing a reflective essay.

**Fig. 1:**
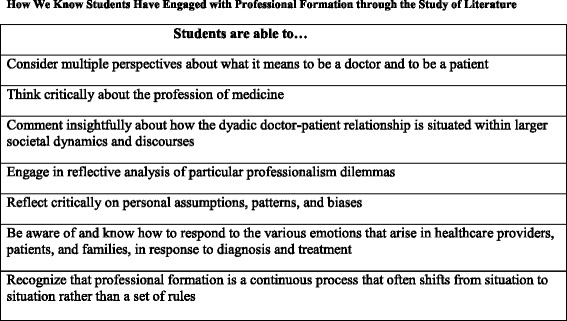
How we know students have engaged with professional formation through the study of literature

Assessment of the understanding of professionalism that students glean from literature will be better achieved through qualitative, narrative means [69]. Longitudinal evaluation by instructors, to allow for the maturation of professional identity, that examines both individual and collaborative student writing and creative projects [70] reflecting on professional formation issues and dilemmas, as well as narrative self-assessment of professional development might be considered according to criteria listed in Fig. 1. In considering such student work, pedagogical theory in the humanities suggests that what is important is transparency in how the student *thinks*, rather than the specific nature of the conclusions they reach [71, 72].

Following this line of reasoning, we suggest that projects, essays, and other relevant products should be examined for their ability to make students’ thinking about professionalism formation and dilemmas visible and plain. This might mean, for example, attending to how a student both develops and questions an argument, considers multiple perspectives, understands emotional sequelae for both self and others, and has some sense of the relevant cultural, historical, familial, and personal factors implicated. Further, research suggests that professionalism decisions in medicine are highly context dependent [73], are influenced by a wide range of considerations, and are surprisingly shifting and malleable depending on the input of peers [74]. These findings suggest that assessment of professionalism cannot be global and general, but must be situation specific.

Since it is impossible to anticipate all professionalism dilemmas, it is particularly important to nurture habits of mind such as are outlined above that can be brought to bear on unique clinical encounters. For example, Kuper suggests that students’ increasing emotional awareness, self-reflection, and capacity to grasp ambiguity might be considered as proxy outcomes for actual patient interaction skills [75]. Here again, such qualities cannot be measured through a Likert scale, but might be explored through an evaluative process that explores students’ growth on these dimensions, and explores how these qualities can be translated into real-world situations. Charon talks about “narrative evidence”, or the insights and sensibility offered through careful attentiveness to the patient’s story [76]. We might do well to refer to this concept in assessing what medical students learn from exposure to literature – i.e., what have they discovered about how to access the person of the patient in a medical interview? How has their understanding evolved regarding the ways in which a patient’s cultural background, class, family and community affect her response to illness? Within this framework, evaluation of learners might best be understood as a kind of conversation between faculty and student rather than a definitive, top-down assessment.

Charon also points out that the true metrics of success have to do with clinicians’ attitudes, behavior and interactions in the clinical arena, and the effects these have on their patients [77]. Following Charon’s lead, we suggest that the gold standard of professionalism is patient and family assessment of these dimensions of care in their student doctors. By this we do not mean yet more patient satisfaction measures of learners. Rather, time-consuming as it would be, obtaining narrative responses from patients and families about how they experience the trustworthiness, respectfulness, non-judgmentalism of learners, their capacity to listen and care and to demonstrate compassion in action by thinking outside the box, would be a truly meaningful form of assessment. Such an approach is an essential way to reveal to what extent nuanced scrutiny of stories, poetry, and essays by patients and physicians affects the way learners interact with and behave toward actual patients and families. By making such inquiries of patients and their family members, we would learn how students translate the professionalism values, attitudes, and interactive skills they have discovered in literature into each unique of clinical encounters.

## Conclusion

In summary, we suggest that literature is an essential element of medical education that, through the method of close reading, contributes intellectual inquiry, emotional awareness, sociocultural context, and a countercultural perspective to questions regarding medical professionalism. Narrative and storytelling broaden and make more complex the ethical context of care provided by students and faculty. They assist learners in rigorously and feelingly examining, in specific evocative contexts, what it means to be a doctor in relationship with patients and families within a framework of larger social dynamics and discourses. Literature can deepen the understanding of medical professionalism, as many medical educators desire; but it cannot simultaneously promote assessment practices that rely on facile quantitative behavioral responses. If medical education can not only tolerate but embrace the opportunity to challenge the assumptions and beliefs its learners hold about the profession, literature has much to offer professionalism formation.

## Footnotes

1 Some examples of short stories and poems that encourage self-examination and broader thinking include: "Laundry" by Susan Mates, "Touching" by David Hellerstein, "Carnal Knowledge" by Danny Abse, "Skin for Ricky" by DL Scheidermayer, "Baptism by Rotation" by Mikhail Bulgakov, "Imelda" by Richard Selzer, and "Talking to the Family" by John Stone. While doctors are not the only or the most important storytellers, such texts are especially useful entry points for medical learners uncertain as to the value of studying narratives as part of their medical education.
